# A Comparative Survey on Daily Health Habits Among iPhone and Android Smartphone Users

**DOI:** 10.1177/15598276241268195

**Published:** 2024-07-27

**Authors:** Matthias N. Ungerer, Christoph Gumbinger

**Affiliations:** 1 Department of Neurology, Heidelberg University Hospital, Heidelberg, Germany (MNU, CG)

**Keywords:** habits, healthy lifestyle, mobile applications

## Abstract

**Background:** With the increasing use of smartphone-based lifestyle interventions, it is important to understand whether people’s preferred operating system (iOS or Android) is linked to their lifestyle habits. **Purpose:** Our goal was to determine whether the choice of an iOS- or Android-powered smartphone independently affected daily health habits. **Methods:** We recruited participants for an online survey using the CloudResearch® Connect™ platform. The survey collected basic sociodemographic data, information on the participant’s primary smartphone operating system, and the HLPCQ to estimate daily health habits. **Results:** Of the 195 participants, 54.4% identified as Android users. We found that iOS users tended to be younger, had higher estimated annual household incomes, and had higher levels of education. However, we found no statistically significant link between the operating system and the HLPCQ total score or any of its subscales. Instead, age, employment status, and estimated annual household income were found to be more strongly associated with daily health habits. **Conclusion:** This study did not find a significant association between the smartphone operating system and daily health habits as measured by the HLPCQ. The findings suggest that future smartphone-based lifestyle interventions should instead focus on established sociodemographic factors linked to lifestyle habits.

‘Our findings reveal a significant correlation between lifestyle choices and income’.

## Introduction

Smartphone-based applications (apps) have become an integral part of our daily lives, oftentimes by influencing our lifestyle habits. A smartphone’s operating system (OS) may play a significant role in shaping these habits.

Over 99% of the market share of operating systems is primarily divided between iOS and Android.^1^ Thus, this paper investigates the differences in lifestyle habits between iOS and Android users. Health-promoting apps are offered on both smartphone operating systems, covering topics ranging from mental health to smoking cessation.^2,3^ Both platforms offer many of the same apps, as well as others developed specifically for Android or iOS.

Differences in user profiles of Android- and iOS-powered smartphones – based on factors such as income, socioeconomic status, and age – have been suggested in several publicly available consumer polls. For example, iOS users based in the United States tend to consist of young adults who earn high incomes.^4^ Other polls have suggested that the behaviour patterns of iOS and Android users may also differ in terms of app usage, purchasing habits, brand loyalty, and social behaviour.^5,6^ However, the relationship between smartphone OS and lifestyle habits remains largely unexplored. Differences in adherence to smartphone-based smoking cessation interventions have previously been reported^7^; however, relevant differences in personality types have not been found in another study.^8^

Researchers must be aware of potential differences in lifestyle habits and user preferences for health-promotion interventions. It is important to understand if smartphone OS is associated with differences in users’ daily health habits because this information can help to create tailored apps that can optimise the effectiveness of health interventions and improve user adherence.

The main goal of this study is to determine whether the use of an iOS- or Android-powered smartphone has any association with differences in daily health habits. We conducted an online survey using the Healthy Lifestyle and Personal Control Questionnaire (HLPCQ) to explore differences in dietary habits, physical activity, sleep quality, and social and mental well-being based on the OS that the participants used. It is possible to explain the association between a person’s preferred smartphone OS and their daily health habits in one of 2 ways. First, it could be due to the ‘selection effect’, whereby people who already have a healthier lifestyle are more likely to choose a certain OS. Alternatively, it could be a result of the ‘treatment effect’, where using a specific OS leads to adopting healthier daily habits.

By understanding the possible association, we can gain insight into how technology affects our daily habits and well-being. Additionally, these findings could inform the design of personalised health interventions delivered through smartphones.

Through this comparative analysis, we aim to contribute to the growing body of research on the intersection of technology and lifestyle habits, providing valuable insights for researchers, policymakers, and app developers in the health and technology sectors.

## Methods

### Study Design

A cross-sectional study was performed. We used an online survey with participants recruited from the United States. Participants were recruited on the platform CloudResearch^®^ Connect™ and redirected to the survey on Google Forms™. We aimed to include a quota of 50% of participants who primarily used either Android or iOS on their smartphones. All participants were required to be at least 18 years old. We did not have any additional preselection criteria. All participants were compensated with a participation fee (equivalent to an hourly wage of 18 dollars), which was in line with industry standards. Participation in the survey was voluntary, and all data was collected anonymously and remained so throughout the study. At no point was collected data traceable to an individual survey participant. Participants were informed of the purpose of the study and that the results would be published in a peer-reviewed scientific journal. We consulted with the local ethics committee before the study commenced. It was concluded that official ethics approval was not necessary since data was collected anonymously, and it was not to be expected that the participants of the survey would be exposed to psychological or physical stress because of their participation.

### Design of the Online Survey

The questionnaire consisted of 2 sections with a total of 32 questions and included a description of the research project provided to participants (see the Supplemental Materials for a copy of the questionnaire/project description). Section 1 asked participants which OS they predominately used and included questions on the sociodemographic profiles of patients with a known association with lifestyle habits. These included questions on age, gender, educational degree, household income (based on categories of income quintiles in the United States in 2021), and employment status. All questions in Section 1 were presented in a multiple-choice format, except for the question on the age of participants, which required a short-written answer. Section 2 included 26 questions from the validated HLPCQ questionnaire that assesses current daily health habits. The HLPCQ was developed by Darviri et al. specifically for the evaluation of lifestyle interventions.^9^ The questionnaire uses positively phrased questions and assesses the participants’ health empowerment. It focuses on daily routines related to diet, physical exercise, and mental well-being. It was uniquely designed to assess the effect of health-promotion interventions on participants’ daily activities. The HLPCQ has since been validated in international populations and has been used in several studies to assess daily health habits.^10–14^ Participants were asked how often they engaged in positive lifestyle habits. All questions in the HLPCQ are answered using a four-point Likert scale (1 – Never or Rarely, 2 – Sometimes, 3 – Mostly, and 4 – Always). The questions in the HLPCQ are organised into 5 subscales: dietary healthy choices (7 items), dietary harm avoidance (4 items), daily routine (8 items), organised physical exercise (2 items), and social and mental balance (5 items). The total score can range from a low of 26 to a high of 104, with higher scores indicating healthier daily routines. Overall, we estimated that the average participant would require approximately 5 minutes to complete the survey based on a test run by an independent researcher. The duration of the survey was advertised to be under 10 minutes. The survey was hosted on Google Forms™. Participants were directed to the survey by URL from the Connect™ Website and were not required to identify or specify any personalised or identifiable data. Answering all questions in the survey was mandatory to receive a completion code at the end of the survey.

### Sample Size Calculation and Statistical Analysis

We estimated the necessary sample size up front to detect significant differences between the two means of the HLPCQ total score based on available data from the original validation study. The sample size necessary to detect a difference in the means corresponding to half the expected standard deviation (5.92 points) with a power of 80% and a significance level of 5% (two-sided) was 128 total participants, assuming equal group sizes (64 in each group). Statistical analysis was performed using IBM SPSS Statistics 28.0.1. Conventional statistical methodologies were employed for the data description. The mean values, standard deviations, and percentages were approximated to the nearest tenth. The independent *t* test was utilised to discern differences in mean values, whereas the chi-squared test was employed for identifying group differences in non-continuous variables. A *P* value of 0.05 was established as the threshold for statistical significance. Before conducting the statistical analysis, the dataset was examined for duplicate entries.

## Results

A total of 195 individuals (with 20 people in a pilot study) participated and completed a survey on 3 and 4 January 2024. All survey questions were mandatory, and there were no incomplete responses. After verifying that there were no duplicate entries, we included all the surveys that were submitted in the analysis. Participants took an average of 4 minutes and 58 seconds to complete the survey. In all, 100 participants rated the study with 4.9 out of 5 stars, indicating high satisfaction. The participants’ ages ranged from 19 to 73 years old. The mean age of the participants was 36.8 ± 10.4 (median 36), and 55.9% were male. Most participants worked full-time (60.0%) or part-time (11.3%) were self-employed (9.7%), while 14.9% were unemployed. Most participants (67.2%) had a college degree (associate/bachelor’s degree or higher). The majority of participants were considered ‘middle-class’, with 71.9% earning an estimated annual household income between $28,000 and $150,000, while 16.9% earned less than $28,000 and 11.3% more than $150,000.

A total of 106 (54.4%) participants were identified as mainly Android users, while 89 (45.6%) used iOS. The mean HLPCQ total score was 63.3 ± 11.6. The mean scores for the HLPCQ subscales were 16.8 ± 3.7 for dietary health choices, 10.1 ± 2.7 for dietary harm avoidance, 19.2 ± 5.0 for daily routine, 4.8 ± 2.0 for organised physical exercise, and 12.4 ± 2.7 for social and mental balance.

### Differences Between iOS and Android Users

Tables 1 and 2 provide a detailed comparison of demographic data and HLPCQ results. Our analysis revealed that iOS users were typically younger (35.0 ± 9.2 years compared to 38.3 ± 11.1 years for Android users) and had a higher level of education (14.6% of iOS users had an advanced college degree compared to 8.5% of Android users). Additionally, iOS users had a higher estimated annual household income. We did not find any significant differences in terms of gender distribution or employment status, although full-time employment was more common among iOS users, and part-time employment, self-employment, and unemployment were slightly more prevalent among Android users. Our analysis showed that there was no association between the OS used and the HLPCQ total score or any of its subscales, as confirmed by both correlation and regression analyses.

**Table 1.** Comparison of Sociodemographic Profiles for iOS and Android Users.

	iOS (n = 89)	Android (n = 106)	Test Used	T or Chi-Squared Value	*P* value
Age (mean, SD)	35.0 ± 9.2	38.3 ± 11.1	*t* test	2.224	**0.027**
Male (n, %)	45 (50.6%)	64 (60.4%)	Chi-squared	1.891	0.169
Employment status (n, %)			Chi-squared	5.794	0.215
- Full-time	59 (66.3%)	58 (54.7%)			
- Part-time	6 (6.7%)	16 (15.1%)			
- Self-employed	7 (7.9%)	12 (11.3%)			
- Temp/Contract	5 (5.6%)	3 (2.8%)			
- Unemployed	12 (13.5%)	17 (16.0%)			
Highest education (n, %)			Chi-squared	12.409	**0.015**
- Less than a high school diploma	0 (0%)	1 (0.9%)			
- High school diploma	7 (7.9%)	19 (17.9%)			
- Some college, no degree	11 (12.4%)	26 (24.5%)			
- Associate or bachelor’s degree	58 (65.2%)	51 (48.1%)			
- Advanced college degree	13 (14.6%)	9 (8.5%)			
Annual household income (n, %)			Chi-squared	19.708	**<0.001**
- <$28,000	8 (9.0%)	25 (23.6%)			
- $28,000–$55,000	16 (18.0%)	35 (33.0%)			
- $56,000–$90,000	27 (30.3%)	17 (16.0%)			
- $90,000–$150,000	23 (25.8%)	22 (20.8%)			
- >$150,000	15 (16.9%)	7 (6.6%)

**Table 2.** Comparison of HLPCQ Scores in iOS and Android Users.

Score	Operating System	Mean	Standard Deviation	t value	*P* value (*t* Test)
Total score	Android	63.50	11.929	0.314	0.754
	iOS	62.98	11.151		
Subscales					
- Dietary health choices	Android	16.94	3.598	0.660	0.510
	iOS	16.60	3.744		
- Dietary harm avoidance	Android	10.28	2.950	1.06	0.291
	iOS	9.87	2.473		
- Daily routine	Android	19.23	4.923	0.096	0.923
	iOS	19.16	5.083		
- Organised physical exercise	Android	4.71	1.971	−0.977	0.330
	iOS	4.99	2.037		
- Social and mental health	Android	12.34	2.911	−0.080	0.936
	iOS	12.37	2.465		

### Association Between Sociodemographic Profiles and HLPCQ Scores

**Figure 1.**
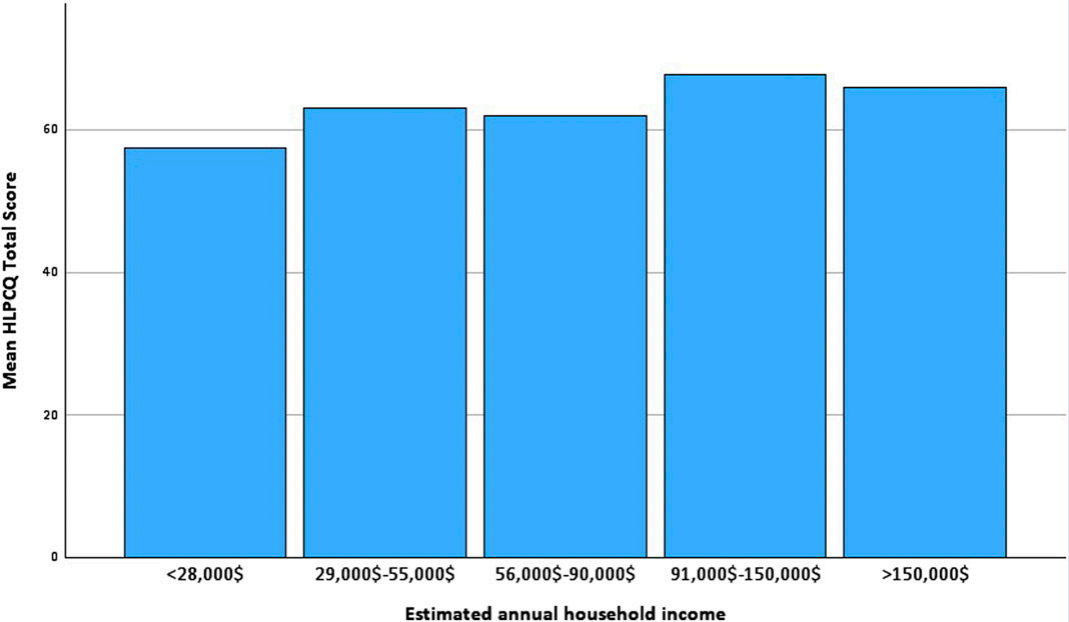
HLPCQ total score by estimated annual household income.

The total score for the HLPCQ was found to be strongly correlated with age (*P* = 0.004), employment status (*P* = 0.003), and estimated annual household income (*P* < 0.001). However, no significant correlations were found between the total score and gender or highest educational degree. We observed higher scores in the subscale of organised physical exercise in males (*P* = 0.014) as the only association of gender with any of the subscales, and the highest educational degree was not correlated with any of the subscales. The results of the multivariate linear regression analysis for all sociodemographic factors are shown in Table 3. Additionally, multivariable regression analysis for the 3 relevant variables confirmed that age, employment status, and annual household income were independent predictors of the HLPCQ total score. Older age was found to be associated with higher total scores, mostly due to higher scores in the subscales of dietary harm avoidance (*P* = 0.001) and daily routine (*P* = 0.008). When the participants were divided into 2 age groups, regression analysis and correlation analysis showed similar associations with the total and subscale scores. Being unemployed or employed part-time was associated with the lowest HLPCQ total scores. Correlation analysis showed that this was due to lower scores for healthy dietary choices and daily routine, and it was most pronounced in unemployed participants. A higher estimated annual household income was found to be a predictor of higher HLPCQ total scores. The highest scores were found in the $91,000–$150,000 bracket, whereas the lowest scores were found in the below $28,000 category (Figure 1). The strongest correlations were found with the subscales of daily routine (*P* < 0.001), organised physical exercise (*P* = 0.004), and social and mental balance (*P* = 0.018).

**Table 3.** Determining the Predictive Factors for HLPCQ Total Scores Through Multivariate Linear Regression Analysis.

	Regression Coefficient	Lower CI	Upper CI	*P* value
OS	−1.263	−4.663	2.137	0.465
Age	0.177	0.017	0.336	**0.030**
Gender	−2.160	−5.353	1.033	0.184
Employment status	0.987	−0.124	2.098	0.081
Highest education	0.283	−1.714	2.279	0.780
Annual household income	1.784	0.367	3.202	**0.014**

Abbreviations: CI, confidence interval; OS, operating system

## Discussion

### Main Findings

#### 1. iOS Users Were Younger, Had a Higher Educational Degree, and Had a Higher Estimated Annual Household Income

Our findings on the sociodemographic profiles of iOS and Android users are in line with previously reported data. Most data available on this subject has been derived from non-scientific consumer surveys. However, our findings align with previously reported data, as the study revealed that iOS users were younger and identified more often as female.^5,15,16^

#### 2. No Association Between Smartphone OS and HLPCQ Total Score or Any of Its Subscales

We found that the type of smartphone OS was not associated with a higher HLPCQ score in the sample studied. The results consistently showed no association between OS and the subscales of the HLPCQ, leading to the conclusion that there was no direct connection between the OS a person used and their lifestyle habits. Importantly, the results of the study indicate that there is no need to adapt lifestyle apps to a specific OS. Rather, simultaneous development of lifestyle intervention apps is possible for both the Google Play Store and the Apple App Store. However, independent predictors for lifestyle habits (such as estimated annual household income) were associated with HLPCQ scores and smartphone OS, which suggests that associations between OS and lifestyle habits could be found in smaller populations as a confounding effect. Researchers should be aware of this possibility when conducting future smartphone-based lifestyle interventions. Developers of smartphone-based health interventions should consider designing apps specifically for target populations based on their socioeconomic composition.

#### 3. Age, Employment Status, and Estimated Annual Household Income Were Identified as Predictors of the HLPCQ Total Score

We were able to confirm several known socioeconomic characteristics as predictors of daily healthy habits. Age was an important predictor. We found that older age was associated with higher HLPCQ total scores, mainly due to higher scores in the subscales for daily routine and dietary harm avoidance. After dividing the participants into 2 age groups (the cut-off age was 40), we confirmed the same results upon repeating the analysis. Both these aspects of daily habits would be expectedly more prevalent in the older group, who are likely to prioritise regular meals and avoid fast food and soft drinks when compared to younger participants. This is partly because the HLPCQ focuses on daily healthy habits and less on categories such as intensive physical exercise, in which younger individuals are expected to have higher scores. Employment status and estimated annual household income are also known predictors of a healthy lifestyle. As expected, a higher socioeconomic status (i.e. higher household income and working full-time or being self-employed) predicted higher HLPCQ scores. Higher income has been associated with higher rates of regular physical exercise,^17^ while income inequality may be detrimental to social well-being.^18^ Previous studies have suggested that regular exercise is more common among high-income and full-time employed individuals compared to low-income and part-time employed individuals.^19^ Nutrition knowledge and diet are also associated with household income. In developed countries, the knowledge of a healthy diet and healthy shopping behaviour is higher among individuals with better financial status.^20,21^ Unemployment, in particular, has been associated with poor dietary habits.^22^ In our study, we found no significant correlation between household income and the subscales of the HLPCQ related to dietary habits. This could be the case because the HLPCQ does not assess nutrition knowledge or shopping behaviour but rather focuses on habits associated with daily eating routines. Regression analysis suggested a linear progression of HLPCQ scores with increasing household income in our study. However, participants who identified with the above $150,000 annual income group had slightly lower scores than those in the $91,000–$150,000 group, suggesting a ceiling effect in the relationship between household income and HLPCQ scores. It is necessary to conduct additional research to better understand the relationship between income and daily health habits. Our findings reveal a significant correlation between lifestyle choices and income. This information can assist researchers in designing customised smartphone-based interventions, which can effectively target people with lower socioeconomic status. These interventions can serve as an alternative to traditional health apps and can prove to be beneficial for individuals who are harder to reach.

### Implications of Our Study

We did not find sufficient evidence that smartphone OS was a predictor of lifestyle habits using the HLPCQ questionnaire. This suggests that the preference for a particular OS may not have influenced lifestyle habits independently, as measured by the HLPCQ questionnaire. However, researchers must be aware that sociodemographic profiles influence lifestyle behaviour independently, which can be relevant when designing apps for targeted intervention studies (and, therefore, adjusting for these factors). It is recommended that researchers determine the socioeconomic profile and preferences of the targeted population before designing an intervention app.

### Strengths

After thorough plausibility checks, the data quality appeared satisfactory. The average time taken to complete the survey aligns with our initial estimate based on an independent third-party tester. Notably, the HLPCQ total score in our study was 63.3 ± 11.6, closely mirroring the 64.4 ± 11.8 score in the original validation study of the questionnaire, lending credibility to our results.^9^ The study had a participant count that exceeded the required sample size, ensuring sufficient data to effectively address the research question. The parameters we compiled for socioeconomics accurately represent the US population and also challenge a significant selection bias.

### Limitations

Several limitations should be considered when interpreting our results. The nature of paid online surveys inherently introduces the potential for self-selection bias, as participation is voluntary. It is important to note that the quick recruitment process and short duration of the study made it unlikely that the participants were preselected. Despite this, it is possible that older seniors who may have unhealthy lifestyle habits were not well represented in our sample group.^23^ As our survey was conducted online on a paid platform, our participants were preselected on the basis of only those willing to participate in surveys. Our participant pool was exclusively from the United States, where iOS is more prevalent than Android, which alternatively holds over 70% of the global smartphone market share and is particularly popular in lower-income nations.^1,5^ Consequently, in regions where iOS usage is predominantly among upper-class households, the choice of operating system could be more strongly linked to socioeconomic status, leading to a greater confounding effect. The survey’s format inherently limited the information we could gather about participants. For example, a more detailed inventory of social influencing factors, such as access to preventative medical measures and insurance status, was not collected and could be examined in future studies on the association between OS and healthy lifestyles. One significant limitation is the sample size. These findings should be confirmed in future larger studies. We also cannot rule out the possibility that the choice of operating system could be linked to more specific lifestyle behaviours that were not all accounted for in this survey, such as more vigorous exercise. Further research could investigate whether certain behaviours (e.g. daily step count or preference for particular physical activities) are associated with smartphone OS. However, the HLPCQ was specifically designed to evaluate lifestyle interventions and to provide a comprehensive and representative assessment of various aspects of daily health habits.

## Conclusion

The findings of this study did not identify smartphone operating systems as standalone predictors of daily health habits. As an exploratory study, these results should be validated in future research with larger participant samples. For those conducting smartphone-based lifestyle interventions, it would be beneficial to focus on the established sociodemographic factors linked to lifestyle habits when designing future health-promotion interventions.

## Supplemental Material

Supplemental Material - A Comparative Survey on Daily Health Habits Among iOS and Android Smartphone Users Supplemental Material for A Comparative Survey on Daily Health Habits Among iOS and Android Smartphone Users by Matthias N. Ungerer and Christoph Gumbinger in American Journal of Lifestyle Medicine

## Data Availability

Data can be made available upon reasonable request.*
