# Supplementary material for: A Comparative Survey on Daily Health Habits Among iPhone and Android Smartphone Users
Source: Am J Lifestyle Med. 2024 Jul 27:15598276241268195. Online ahead of print. doi: 10.1177/15598276241268195 (PMC11562268; doi:10.1177/15598276241268195)
Supplement: Supplemental Material - A Comparative Survey on Daily Health Habits Among iOS and Android Smartphone Users [file sj-pdf-1-ajl-10.1177_15598276241268195.pdf]

A Comparative Survey on Daily Health Habits Among iOS and Android Smartphone Users

The aim of this survey is to determine whether healthy daily habits are more common in IOS or Android smartphone users using the standardized HLPCQ questionnaire. The results could be relevant for future smartphone-based health interventions.

The surveys will be collected and stored anonymously. The results will be published in a peer-reviewed scientific journal. Individual surveys will be deleted once data analysis has been completed.

Section 1 includes questions on demographic data (gender, age, household income, employment status etc.). Section 2 includes questions from the HLPCQ questionnaire.

Estimated completion time: <10 minutes.

* Indicates required question

1. **What operating system is installed on your smartphone or which do you use most frequently if you have more than one smartphone?** *

   Mark only one oval.

   - ◯ Android (Samsung, Google pixel, Motorola, ASUS, Xiaomi, etc.)
   - ◯ IOS (Apple)

2. How old are you (in years) *

   ____

3. **What is your gender** *

   Mark only one oval.

   - ◯ Male
   - ◯ Female

4. **What is your employment status?** *

Mark only one oval.

- Full-time employment
- Part-time employment
- Self-Employment
- Temporary/contract/freelancing
- Unemployed

5. **What is your highest educational degree?** *

Mark only one oval.

- less than a high school diploma or equivalent
- high school diploma
- some college but no degree
- associate or bachelors degree
- advanced degree such as a master's degree, professional degree or doctoral degree

6. **What is your estimated yearly household income?** *

Mark only one oval.

- <28,000$
- 28,000$-55,000$
- 56,000$-90,000$
- 91,000$-150,000$
- >150,000$

The Healthy Lifestyle and Personal Control Questionnaire (HLPCQ)

7. **Are you careful about how much food you put on your plate** *

1 - Never or rarely 2- Sometimes 3- Mostly 4- Always

Mark only one oval.

	1	2	3	4	
Nev	◯	◯	◯	◯	Always

8. **Do you check the food labels before buying a product** *

1 - Never or rarely 2- Sometimes 3- Mostly 4- Always

Mark only one oval.

	1	2	3	4	
Nev	◯	◯	◯	◯	Always

9. **Do you calculate the calories of your meals** *

1 - Never or rarely 2- Sometimes 3- Mostly 4- Always

Mark only one oval.

	1	2	3	4	
Nev	◯	◯	◯	◯	Always

10. **Do you limit fat in your meals** *

1 - Never or rarely 2- Sometimes 3- Mostly 4- Always

Mark only one oval.

	1	2	3	4	
Nev	◯	◯	◯	◯	Always

11. **Do you cook your own food** *

1 - Never or rarely 2- Sometimes 3- Mostly 4- Always

Mark only one oval.

	1	2	3	4	
Nev	◯	◯	◯	◯	Always

12. **Do you prefer organic foods** *

1 - Never or rarely 2- Sometimes 3- Mostly 4- Always

Mark only one oval.

	1	2	3	4	
Nev	◯	◯	◯	◯	Always

13. **Do you prefer whole-wheat products** *

1 - Never or rarely 2- Sometimes 3- Mostly 4- Always

Mark only one oval.

	1	2	3	4	
Nev	◯	◯	◯	◯	Always

14. **Do you avoid eating packaged- or fast-food** *

1 - Never or rarely 2- Sometimes 3- Mostly 4- Always

Mark only one oval.

	1	2	3	4	
Nev	◯	◯	◯	◯	Always

15. **Do you avoid soft drinks** *

1 - Never or rarely 2- Sometimes 3- Mostly 4- Always

Mark only one oval.

	1	2	3	4	
Nev	◯	◯	◯	◯	Always

16. **Do you avoid eating when stressed or disappointed** *

1 - Never or rarely 2- Sometimes 3- Mostly 4- Always

Mark only one oval.

	1	2	3	4	
Nev	◯	◯	◯	◯	Always

17. **Do you avoid binge eating when you are out with friends** *

1 - Never or rarely 2- Sometimes 3- Mostly 4- Always

Mark only one oval.

	1	2	3	4	
Nev	◯	◯	◯	◯	Always

18. **Do you eat your meals at the same time each day** *

1 - Never or rarely 2- Sometimes 3- Mostly 4- Always

Mark only one oval.

	1	2	3	4	
Nev	◯	◯	◯	◯	Always

19. **Are you careful about not missing a meal each day** *

1 - Never or rarely 2- Sometimes 3- Mostly 4- Always

Mark only one oval.

	1	2	3	4	
Nev	◯	◯	◯	◯	Always

20. **Do you eat a good breakfast** *

1 - Never or rarely 2- Sometimes 3- Mostly 4- Always

Mark only one oval.

	1	2	3	4	
Nev	◯	◯	◯	◯	Always

21. **Do you sleep at the same time each day** *

1 - Never or rarely 2- Sometimes 3- Mostly 4- Always

Mark only one oval.

	1	2	3	4	
Nev	◯	◯	◯	◯	Always

22. **Do you follow a scheduled program for your daily activities** *

1 - Never or rarely 2- Sometimes 3- Mostly 4- Always

Mark only one oval.

	1	2	3	4	
Nev	◯	◯	◯	◯	Always

23. **Do you eat breakfast at the same time each day** *

1 - Never or rarely 2- Sometimes 3- Mostly 4- Always

Mark only one oval.

	1	2	3	4	
Nev	◯	◯	◯	◯	Always

24. **Do you eat lunch at the same time each day** *

1 - Never or rarely 2- Sometimes 3- Mostly 4- Always

Mark only one oval.

	1	2	3	4	
Nev	◯	◯	◯	◯	Always

25. **Do you eat dinner at the same time each day** *

1 - Never or rarely 2- Sometimes 3- Mostly 4- Always

Mark only one oval.

	1	2	3	4	
Nev	◯	◯	◯	◯	Always

26. **Do you practice aerobic exercise for 20 or more minutes at least 3 times per week** *

1 - Never or rarely 2- Sometimes 3- Mostly 4- Always

Mark only one oval.

	1	2	3	4	
Nev	◯	◯	◯	◯	Always

27. **Do you exercise in an organized manner** *

1 - Never or rarely 2- Sometimes 3- Mostly 4- Always

Mark only one oval.

	1	2	3	4	
Nev	◯	◯	◯	◯	Always

28. **Do you share your personal problems or worries with others** *

1 - Never or rarely 2- Sometimes 3- Mostly 4- Always

Mark only one oval.

	1	2	3	4	
Nev	◯	◯	◯	◯	Always

29. **Do you concentrate on positive thoughts during difficulties** *

1 - Never or rarely 2- Sometimes 3- Mostly 4- Always

Mark only one oval.

	1	2	3	4	
Nev	◯	◯	◯	◯	Always

30. **Do you clear your mind of thoughts and plans for the following day before going to bed** *

1 - Never or rarely 2- Sometimes 3- Mostly 4- Always

Mark only one oval.

	1	2	3	4	
Nev	◯	◯	◯	◯	Always

31. **Do you care about meeting and discussing with your family on a daily basis** *

1 - Never or rarely 2- Sometimes 3- Mostly 4- Always

Mark only one oval.

	1	2	3	4	
Nev	◯	◯	◯	◯	Always

32. **Do you balance your time between work, personal life and leisure** *

1 - Never or rarely 2- Sometimes 3- Mostly 4- Always

Mark only one oval.

	1	2	3	4	
Nev	◯	◯	◯	◯	Always

Thank you for participating in this survey.

Completion code: 5402db1123

This content is neither created nor endorsed by Google.

Google Forms
